# Expression of estrogen and progesterone receptors in papillary thyroid carcinoma

**Published:** 2016

**Authors:** Mohammad-Reza Jalali-Nadoushan, Reza Amirtouri, Ali Davati, Samaneh Askari, Sepideh Siadati

**Affiliations:** 1 Department of Pathology, Shahed University, Tehran, Iran.; 2 Department of Social Medicine, Shahed University, Tehran, Iran.; 3 Department of Pathology, Babol University of Medical Sciences, Babol, Iran.

**Keywords:** Papillary thyroid carcinoma, Estrogen receptor, Progesterone receptor, Prognosis.

## Abstract

**Background:**

Papillary thyroid carcinoma (PTC), occurs mostly in women and sex hormones may play a role in the pathogenesis and clinical course. The objective of this study was to determine the status and prevalence of estrogen and progesterone receptors in PTC with regard to age, gender, tumor size and lymph node involvement.

**Methods:**

Immunohistochemical stains were performed on 92 tissue blocks of PTC for estrogen receptor (ER) and progesterone receptor (PR) expression in tumor cells. Chi-square test and Mann-Whitney U test were used to determine statistical difference using statistical software SPSS.

**Results:**

The mean age of patients was 39.32±1.7 years (range 13-80) with 79(85.9%) women and 13 (14.1%) men. Lymph node involvement was seen in 76.1% of patients. The average tumor size was 3.6±2.21 cm. The rate of ER and PR expression were 46.75% and 5.6%, respectively. ER expression for females was higher than males (P=0.014), but no relation was found between males and females in PR expression (P=0.7). Also there was no statistical difference between ER and PR expression with respect to age, lymph node involvement and tumor size.

**Conclusion:**

Our study showed higher ER expression in females than males with PTC. No relation was found between the expression of these receptors and age of presentation, lymph node involvement and tumor size. Further investigation is required to determine the prognostic importance of ER and PR in PTC.

Thyroid gland neoplasm account for 1% of all cancers (1.5% of malignancies in females and 0.5% in males) and represent the most common endocrine malignancy. During the past decades, its incidence has increased. Eighty percent of these carcinomas are papillary thyroid carcinoma (PTC) (1, 2). The prognosis of PTC is excellent with an indolent course, regional lymph node metastasis and long-term survival (3). It has a female to male ratio of 4: 1 and occurs mostly in young to middle-age adults. The role of sex hormones in the pathogenesis of thyroid disorders has been well documented, acting through B receptors**. **The presence of estrogen and progesterone receptors (ER and PR) on normal thyroid gland tissue may have a role in the development of neoplastic lesions (4-6). It has been found that estrogen can increase the growth, progression and metastasis of PTC (7-10). Also, estrogen may play a more important role in the pathogenesis of PTC in young women (under 25 years of age) than in women 30 years and older (11).

It has been reported that, in all thyroid malignancies, the average age of disease onset in ER positive cases is lower than ER-negative cases. ER positivity has been demonstrated mostly in differentiated thyroid malignancies (12). The objective of this study was to determine the status and prevalence of estrogen and progesterone receptors in PTC with regard to age, gender, tumor size and lymph node involvement.

## Methods

The medical records of patients who underwent thyroidectomy for PTC in the Surgical Pathology Department of Mostafa Khomeini Hospital, Shahed University, Tehran, Iran during 2006 to 2009 were reviewed. Data regarding age, gender, tumor size and lymph node status were retrieved. 

Paraffin-embedded blocks were used to prepare 3 µm thick slides, then sections were deparaffinized in xylene and rehydrated through graded concentrations of ethanol. All slides were incubated in H2O2- methanol solution (1/9) for 10 minutes to inhibit endogenous proxidases. For antigen retrieval, the slides were incubated with EDTA at 120°C, for 15 minutes.

Immunohistochemistry was performed according to the manufacturer's recommendations (Novacastra, UK). Tissue sections were incubated in the blocking serum for 10 min, then with the primary antibody (Novacastra, UK) for 60 minutes at room temperature, followed by 10 minutes of incubation with a biotinylated secondary antibody. The slides were developed using DAB chromogen. Hematoxylin was used for counterstaining. Using light microscopy, the presence of ER (Alpha form) and PR (A form) was scored under high- power (400 x) in 1000 tumor cells. Clone 6F11 which was raised to the full length alpha form of the estrogen receptor molecule and Clone 16 was specific for a region of the N-terminus of the A form of PR. Any presence of ER (Alpha form) or PR (A form) was considered as positive and absence of them as negative result.

Statistical analysis: Quantitative values of the data were presented as mean+SD. Statistical analysis was done using chi-square, independent t-test and Mann -Whitney U test for significant differences between data sets. Pearson correlation was used to check the relation between continues variables. A p-value less than 0.05 was considered statistically significant.

## Results

**Figure 1:**
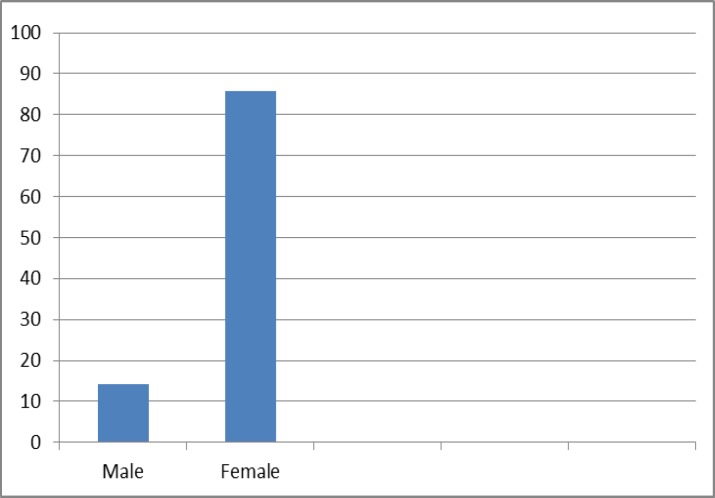
The Percentage of Gender distribution in PTC

This study was performed on tissue blocks of 92 patients with PTC. The mean age of patients was 39.32±1.7 with a range of 13-80 years. There were 79 (85.9%) females and 13 (14.1%) males. Females were older (mean age= 40.3 years) than males (mean age= 33.46 years). However, the observed mean age difference was not statistically significant (P=0.18) (figure 1).

**Figure 2:**
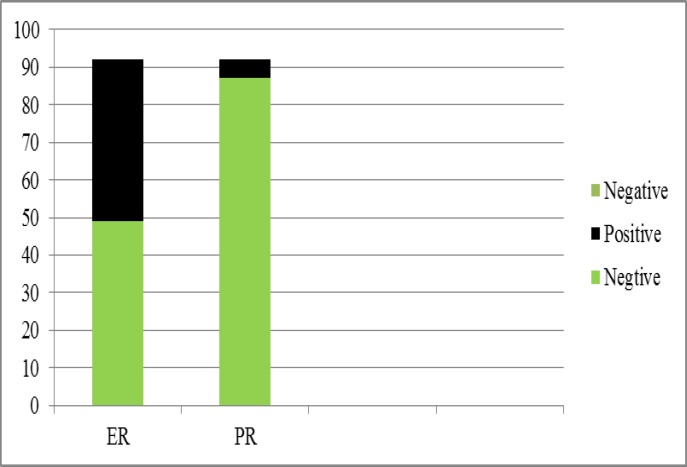
Percentage of ER and PR in specimens

**Figure 3:**
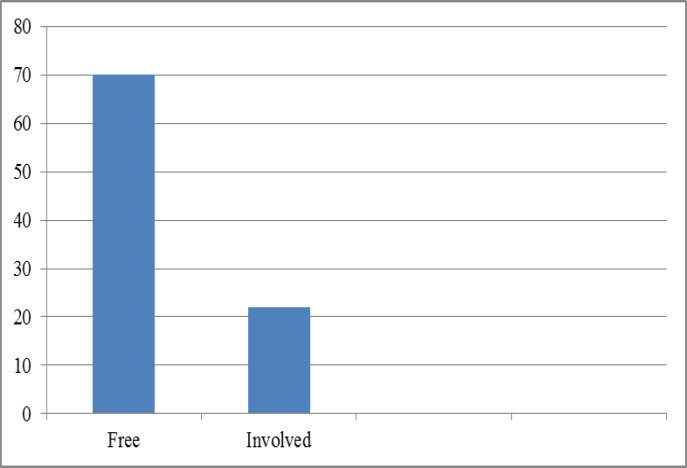
Lymph node involvement

Estrogen receptor (ER) was detected in 46.7% (n=43) of specimens, while this was 5.4% (n=5) for PR (figure 2). Of the 92 patients, 76.1% (n=70) were negative for lymph node metastasis and 22 (23.9%) cases had lymph node involvement (figure 3). The tumor size ranged from 0.5 to 14 cm with a mean range of 3.6±2.21 cm.

In evaluating the possible correlation between ER and PR with different factors, There was no significant correlation between ER expression with age of participants (p=0.627) and the condition was the same for PR (p=0.593).

ER expression was positive in 2 males and 41 females. There was a significant positive correlation between female gender and expression of ER (P=0.014). In contrast, there was no statistical significant variation between males (n=1) and females (n=4) with positive PR expression (p=0.7) (tables 1, 2).

**Table 1:** Gender differences in ER expression

**Gender/ ER**	**Negative**	**Positive**	
Male	11	2	13
Female	38	41	79

**Table 2:** Gender differences in PR expression

**Gender/PR**	**Negative**	**Positive**	
Male	12	1	13
Female	75	4	79

**Figure 4:**
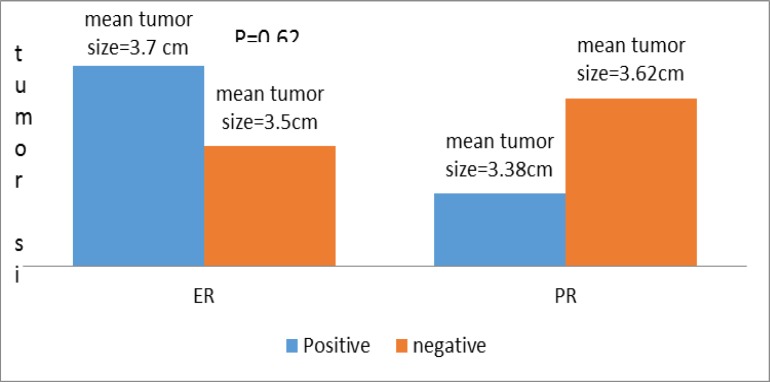
ER and PR expression with respect to tumor size

With regard to the correlation between tumor size and ER and PR expression, tumor size was slightly higher in ER positive cases but not in PR positive expression. Although this difference was not statistically significant (P=0.62) (figure 4).

Considering the invasion potential of tumors, ER expression was positive in 9 cases between patients with nodal metastases (22 patients) although it was insignificant statistically (P=0.53), as for PR (P=0.197) (tables 3, 4).

**Table 3:** ER positivity and lymph node involvement

ER / Lymph node involvement	Positive	Negative
Involved	9	13
Free	34	36

**Table 4:** PR positivity and lymph node involvement

PR / Lymph node involvement	Positive	Negative
Involved	0	22
Free	5	65

Finally, there was a significant correlation between tumor size and age of patients (Pearson correlation =0.27, P=0.01). Most probably this was due to tumor growth by the passing of time.

## Discussion

The present study was conducted on 92 patients with PTC. There are no reported studies investigating the expression of ER and PR in PTC in Iran. Therefore we compared our data with reports from other countries. About half of the 92 cases were ER immunoreactive. There was a relation between ER expression and gender but no relation was found between PR expression and gender. In contrast to our results, Bur et al. showed no relation between ER expression and gender (13). Also, Kansakar et al. showed no difference between ER and PR expression in both genders (14). Such discrepancies may be due to small sample size.

In our study, no relation between lymph node metastasis and ER and/or PR expression was found. This finding is in contrast to other studies suggesting ER positivity to be correlated with metastasis. Rajoria et al. evaluated thyroid cells for the presence of ER and also cell response to estrogen, showing the important role of estrogen in cell division, migration and invasion (15). Considering that their study was performed using Western blot analysis for ER and a cell proliferation assay for estrogen responsiveness, this could explain this difference. 

Vaiman et al. studied thyroid lesions, including PTC, for the detection of alpha and beta ER expression via immunohistochemical staining. Their study showed that ER beta was detectable in thyroid tissue but lacked specificity for discriminating between malignant and benign lesions (16-18). Lack of statistical significance may be due to the absence of a control group in this study. Therefore, we could not compare the diagnostic role of ER in PTC with other thyroid lesions. Also, Vaiman concluded that testing of the ER expression in PTC was not necessary. In their study, ER alpha was undetectable in both benign and malignant lesions (16-18) which was in contrast to our finding that ER alpha was detected in some PTC samples. Kansakar et al. found that the expression of ER and PR in thyroid neoplasms was higher in comparison with normal thyroid tissue, suggesting the role of these hormones in the pathogenesis of thyroid malignancy. This is in agreement with our study (14).

In one study, Inoue et al. compared 10 women under the age of 25 years with 64 women 30 year of age or older who underwent thyroidectomy for PTC. They found higher immunoreactivity for ER in young women, suggesting a promoting role for estrogen (11).

However, in our study of 92 patients (including 79 women) with a mean age of 34, we could not find a relation between age and ER. Our data confirmed that those of Bur et al. reported ER positivity in PTC cases. They could not find any relation between ER expression and age, tumor size, presence of capsular or vascular invasion, lymph node status, or gender (13). Except for the last finding (gender), the other results are similar to our study. 

Hiasa et al. studied 313 thyroid lesions (including 144 thyroid carcinoma) and in contrast to our findings did not find a gender difference with ER immunoreactivity. They concluded that gender differences of ER might be due to a higher serum estrogen level in women. Their findings demonstrated a positive correlation of ER expression with early tumor stage and younger age (12). Based on our data and several studies, ER and PR expression does not appear to have any significant relationship with the type and status of PTC. However, the value of these markers in young women requires further studies.

## Notes

**Funding:** This paper is the result of a medical student thesis and it has been financially supported by Shahed University.

## Citation:

Jalali-Nadoushan MR, Amirtouri R, Davati A, et al. Expression of estrogen and progesterone receptors in papillary thyroid carcinoma. Caspian J Intern Med 2016; 7(3): 183-187.
